# Saccadic body turns in walking *Drosophila*

**DOI:** 10.3389/fnbeh.2014.00365

**Published:** 2014-10-22

**Authors:** Bart R. H. Geurten, Philipp Jähde, Kristina Corthals, Martin C. Göpfert

**Affiliations:** Department of Cellular Neurobiology, Georg-August University of GöttingenGöttingen, Germany

**Keywords:** *Drosophila*, saccades, optic flow, visual acuity, halteres, walking, head body coordination

## Abstract

*Drosophila melanogaster* structures its optic flow during flight by interspersing translational movements with abrupt body rotations. Whether these “body saccades” are accompanied by steering movements of the head is a matter of debate. By tracking single flies moving freely in an arena, we now discovered that walking *Drosophila* also perform saccades. Movement analysis revealed that the flies separate rotational from translational movements by quickly turning their bodies by 15 degrees within a tenth of a second. Although walking flies moved their heads by up to 20 degrees about their bodies, their heads moved with the bodies during saccadic turns. This saccadic strategy contrasts with the head saccades reported for e.g., blowflies and honeybees, presumably reflecting optical constraints: modeling revealed that head saccades as described for these latter insects would hardly affect the retinal input in *Drosophila* because of the lower acuity of its compound eye. The absence of head saccades in *Drosophila* was associated with the absence of haltere oscillations, which seem to guide head movements in other flies. In addition to adding new twists to *Drosophila* walking behavior, our analysis shows that *Drosophila* does not turn its head relative to its body when turning during walking.

## Introduction

As most insects lack stereoscopic vision (Land, 1999) to gauge the distance of surrounding objects, visual cues created by self-motion must be exploited. Locusts and mantids, for example, perform peering movements with their heads to deduce the distance of objects from the resulting motion parallax (Kral and Poteser, 1997). Motion parallax is also exploited by wasps that perform curved learning flights to remember the sites of their nests (Zeil, 1993, 1996). In more general terms, any movement of the animal's head will create an image shift on the retina—a phenomenon known as optic flow (Gibson et al., 1955). During translations, close objects will travel faster across the retina than distant ones, providing distance information, whereas no such information can be deduced during pure rotations when all objects travel across the retina with equal speeds (Koenderink and Doorn, 1987).

To facilitate distance estimation, insects thus should (i) separate translational movements from rotations and (ii) turn quickly to reduce the rotation time. Both strategies have been reported for flying *Drosophila* (Heisenberg and Wolf, 1979; Tammero and Dickinson, 2002) as well as other insects (Land, 1973; Collett and Land, 1975; Buelthoff et al., 1980; Zeil, 1986; Geurten et al., 2010), which all seem to structure their locomotion into prolonged phases of predominantly translational movement that are interspersed by fast saccadic turns. The active movement of the head during these saccades was analyzed with varying results (Land, 1973; Geiger and Poggio, 1977), but could be clarified by high-speed observations in freely flying insects (Schilstra and van Hateren, 1998): The head rotates relatively to the body, reducing the saccade duration even further. The role of head body coordination in walking insects is gaining new momentum (Ribak et al., 2009; Kress and Egelhaaf, 2012, 2014a) and questions the information content of the optic flow obtained during walking (Kress and Egelhaaf, 2014b). Nonetheless optic flow has been shown to allow walking *Drosophila* to estimate distances of up to 80 times the length of its body (Schuster et al., 2002). We now tested whether walking *Drosophila* temporally separate rotations from translations and found that this separation is present and that the walking flies perform body saccades.

## Materials and methods

### Walking trajectories

56 male and 57 female adult Canton S wild-type flies were released one by one into circular arenas (43 mm diameter, 3.5 mm height). The arenas were produced using an Ultimaker 3D printer (Ultimaking LTD, Geldermalsen, Netherlands). The lower 1.5 mm of each arena was filled with 1% agarose containing 1% glucose, leaving the upper 2 mm for the flies to walk around. Each arena was illuminated by three Honeycomb LED lamps (IS, Imaging Solutions GmbH, Eningen, Germany), and the flies were filmed from above with a MotionTraveller 500 camera (IS). Movies were recorded using TroublePix software (NorPix Inc., Montreal, Canada) and trajectories were subsequently traced with ivTools (Jens P. Lindemann and Elke Braun, https://toolkit.cit-ec.uni-bielefeld.de/components/tools/ivtools). Only sequences during which the flies did not follow the wall but walked freely through the arena were included in the analysis. The total recording time was about 16 min, yielding half a million frames.

### Prototypical movement patterns

Prototypical movement patterns (PMs) were deduced as described by Braun et al. (2010): Distances between subsequent fly positions were determined as the squared Euclidian distance, and the respective thrust, slip, and yaw velocities were deduced and z-scored individually. To identify the most common velocity combinations, we used two different clustering algorithms, agglomerative hierarchical clustering, and k-means clustering (MacQueen, 1967; Milligan and Cooper, 1987; Murtagh and Contreras, 2012): agglomerative hierarchical clustering (Ward's criterion) was used to narrow down the number of possible PMs. Because hierarchical clustering is only possible for small data sets, the data was chopped into 200 chunks in a round-robin fashion. Less than 20 possible PMs were identified, which were then tested for the whole data set by *k*-means clustering. We tested all PMs between number 2 and 20 and every fifth class between 25 and 50. PMs that describe the data set best were then narrowed down using the quality and the stability of the clustering as operational criteria: quality was calculated as the distance between the PMs divided by their individual density. Stability was assessed by omitting 10, 25, and 50% of the data in a round-robin fashion (step size equaling 2% of the data) to test whether the clustering can be reproduced. For more details on clustering movement trajectories see Braun et al. (2010) and Hofmann et al. (2014).

### Saccades

Saccades were defined using a yaw velocity threshold of 200 deg^*^s^−1^ as the threshold criterion. The peak velocity of each saccade was determined using the “findpeaks” routine of Matlab (R2012b, The Mathworks Inc., Natick, MA, USA). Using these peaks as trigger points, saccades were averaged over a 100 ms time window.

### Head and thorax movements

To simultaneously assess head and body trajectories, flies were filmed while walking through a labyrinth. This labyrinth consisted of two small rectangular boxes (24 x 24 x 3 mm) connected by a 1 cm long 2 x 2 mm tunnel, with a right angle at half its length. Flies were filmed as described above, whereby we used a custom-made planar macroscopic objective to optically resolve thorax and head. Custom-made 3D templates of the fly thorax and head were fitted frame-by-frame to the respective body parts using ivTools (http://www.ivtools.org/ivtools/index.html) to deduce their orientation.

### Retinal images and image shifts

Ommatidial maps were adapted from Petrowitz et al. (2000) (blowfly *Calliphora vicina*), Stürzl et al. (2010) and Seidl (1986) (honey bee *Apis mellifera*), and Buchner (1971) and Dickson et al. (2006) (*Drosophila*). A complete data set for the *Drosophila* eye can be found at http://code.astraw.com/*Drosophila*_eye_map/ (courtesy of Dr. A. Straw). The available map for *Calliphora* is incomplete in that it covers only the frontal part of the eye. Maps were always made for one eye and then mirrored to simulate the opposite eye. Photoreceptor acceptance angles of *Drosophila* were taken from Gonzalez-Bellido et al. (2011), and the corresponding values for *Apis* and *Calliphora* from Laughlin and Horridge (1971) and Smakman et al. (1984), respectively. Acceptance angle data was fitted with a Gaussian. In case of *A. mellifera*, two Gaussians were used for vertical and horizontal acceptance angles (Laughlin and Horridge, 1971). Retinal inputs for panoramic images were calculated by projecting the Gaussians onto the image along the ommatidial axes. To ensure that the entire input of each ommatidium is covered, we extended the Gaussians to five times the standard deviation σ. We than calculated a weighted mean of the panoramic section defined by the Gaussian with the Gaussian filter strength as weights. To rebuild the optical images, we stitched together the Voronoi cells (Lejeune Dirichlet, 1850; Voronoi, 1908) around the ommatidial axes of the eye. To deduce the retinal image shifts that ensue from yaw rotations, we used fifteen full panoramic images of nature scenes (licensed under creative commons by Janne Voutilainen and Aldo Hoeben). Images are shown in Supplementary Figure 1. Ten images show forest scenes and five show close ups of flowers or trees. We also tested ten random images with 3600 x 1800 pixels each and a 1/f^2^ spatial distribution (Field, 1987; van der Schaaf and van Hateren, 1996; Saremi and Sejnowski, 2013) (see Supplementary Figure 2). We then rotated the images by 360° in steps of 0.1° and calculated the resulting retinal image difference (Zeil, 2012) for each ommatidium. The resulting retinal image differences were averaged across ommatidia. The median image difference over different images was calculated for each rotation and each of the three species. For comparison, we employed the same methods to analyze the raw images, averaging over all pixels instead of ommatidia. The median image difference of the raw image between 170 to 180° and −170 to −180° was used to normalize all image difference functions for a given image.

### Haltere movements

To test for haltere oscillations, we replaced one side of the labyrinth with Perspex glass, allowing us to observe the flies from the side. 10 animals, including 5 males and 5 females were filmed while they walked through the labyrinth. Subsequently, the same animals were tethered on their thorax and filmed during fictive walking and flight. To elicit walking, we allowed the flies to grab a small Styrofoam ball, whose removal initiated flight. By touching the legs of the flying flies with the ball, landing behavior was initiated.

## Results

### Walking flies separate translations and rotations

**Figure 1:**
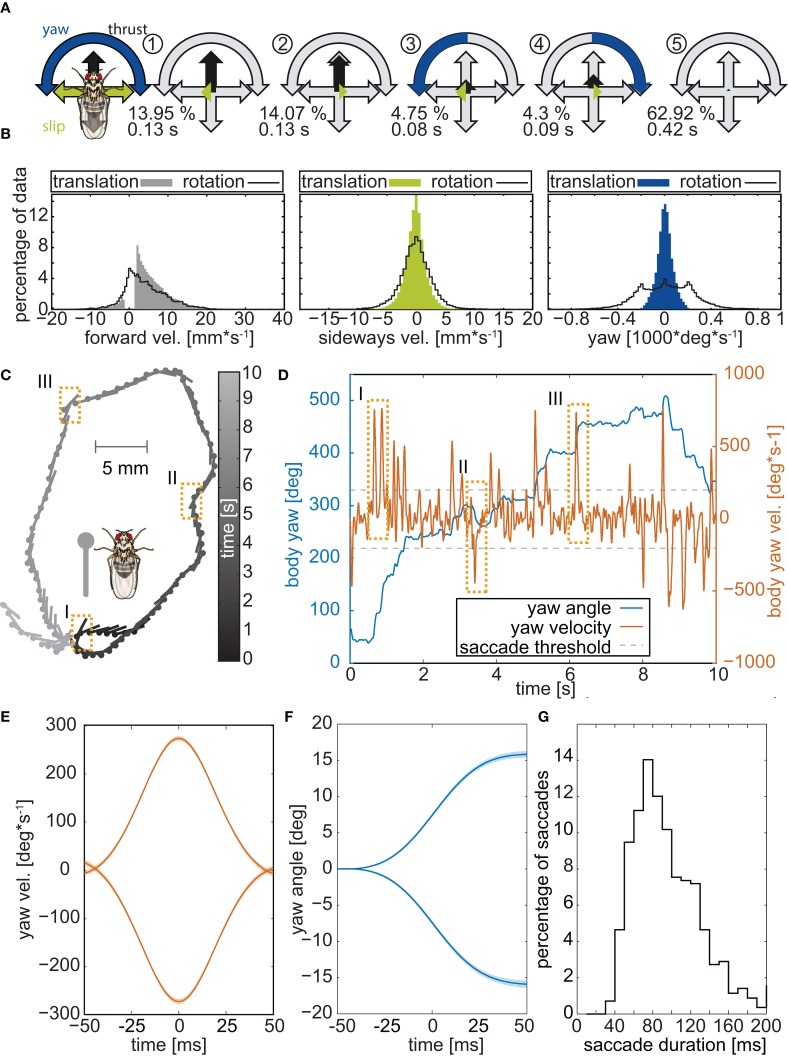
**Body trajectories of walking *Drosophila***. **(A)** Prototypical movement patterns (PMs) of adult *Drosophila* walking freely in a circular arena (56 male 57 female flies, recorded @ 500 fps). Colored arrows highlight the velocity combination that characterizes each PM, and gray arrows indicate the maximum speeds. The color code is given by the legend to the left. For each PM, its respective abundance in the data set is presented in percent, along with its average duration. **(B)** Distributions of the three velocities (thrust, slip, yaw) during translational movements and saccadic turns. Yaw velocities cluster around zero during translations but not during rotations. **(C)** Example of a single trajectory. Circles mark the center of mass of the animal and lines depict the long axis of its body. To facilitate following the trajectory, gray scales indicate time. Dashed boxes highlight three saccades. **(D)** Yaw angles and velocities of the trajectory in **(B)** plotted against time. Orange rectangles mark the three saccades that are highlighted in panel **B**. **(E)** Mean velocity of 1885 individual saccades, identified by using a yaw velocity of 200 deg^*^s^−1^ as the threshold criterion and arranged so that their peak velocity is at 0 ms. Leftward and rightward turns were separated prior to the analysis, yielding velocity profiles for each of them that are virtually mirror-symmetric. **(F)** Corresponding yaw angles documenting that, within the 100 ms time window, the flies turned by on average 15°. **(G)** Distribution of saccade durations in milliseconds.

To test whether walking flies might separate translational and rotational movements, we recorded the trajectories of 103 Canton-S wild-type flies walking freely in an arena at 500 frames per second and screened their body trajectories for reoccurring prototypical movement patters (PMs; Braun et al., 2010). To identify PMs, the respective yaw, slip, and thrust velocities were deduced from the trajectory data, and the most common velocity combinations were extracted using clustering algorithms (see Materials and Methods). Five PMs were identified (Figure 1A), representing translations (PMs 1,2 in Figure 1A), rotations (PMs 3,4), and resting (PM5). Resting (PM5) amounted to 63% of the sampling time, translations (PMs 1,2) for 29%, and only 9% for rotations (PMs 3,4). Consistent with observations on walking *Calliphora*, (Kress and Egelhaaf, 2012, 2014a) walking *Drosophila* showed some residual rotations during translatory phases (Figure 1B), yet the respective rotational velocities were much lower than during phases of turning. Hence, during *Drosophila* walking, (i) translational movements are temporally separated from fast rotations and (ii) translations temporally dominate over rotations.

### Walking *drosophila* perform saccades

A temporal segregation of translational and rotational movements, as revealed by PM analysis, was also seen within single walking trajectories (Figure 1C). Changes in the heading of the flies were associated with rapidly changing yaw angles and sharp yaw velocity peaks (Figure 1D). Selecting heading changes with absolute yaw velocities exceeding 200° per second and using the respective velocity maxima as trigger points, we averaged the yaw velocities for 1140 heading changes of the 103 experimental flies (Figure 1E). Thereby we discarded 3348 slow rotations, whose velocities were below threshold. Average yaw velocities displayed the bell-shaped form that characterizes saccadic eye movements in mammals (Land, 1992; Stanford et al., 2010) and saccadic body turns in insects (Blaj and van Hateren, 2004; Ribak et al., 2009; Kress and Egelhaaf, 2014a). During a saccadic turn, the flies changed their heading on average by about 15° (Figure 1F) within 40 to 120 ms (median 90 ms Figure 1G). These angular heading changes are smaller than those reported for tethered flying *Drosophila* (ca. 90° in 100 ms, Tammero and Dickinson, 2002) or even freely flying *Drosophila* which are nearly twice as fast as tethered animals (ca. 90° in 45 ms, Fry et al., 2003), but close to those of walking blowflies (ca. 15° within 50 ms, Blaj and van Hateren, 2004), which also perform saccades while they walk (Blaj and van Hateren, 2004; Kress and Egelhaaf, 2014a,b).

### Saccadic body turns are not associated with additional head movements

**Figure 2:**
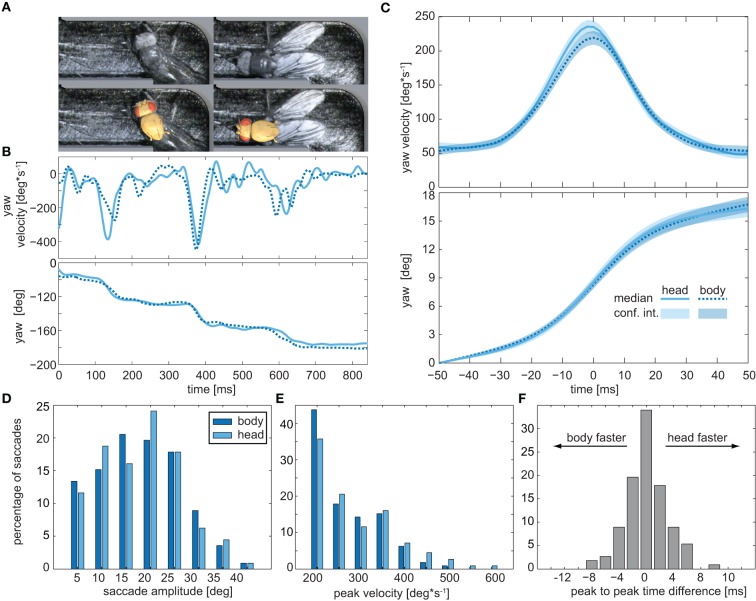
**Head movements during saccadic turns**. **(A)** Snapshots of a fly turning around the right angle in the labyrinth (top) and fitted head and thorax templates (bottom) used to deduce their respective orientations. **(B)** Example trace of the yaw velocities (top) and yaw angles (bottom) of the head (solid blue lines) and body (dashed blue lines) of a fly walking through the labyrinth. Head and body were traced separately by two different investigators (head: B.G., thorax: P.J.). **(C)** Average yaw velocities (top) and yaw angles (bottom) obtained for 114 saccades observed in the labyrinth. Colored areas depict the 95% confidence intervals. **(D)** Histogram of yaw angle amplitudes during saccades (dark blue: body, light blue: head). **(E)** Distribution of the peak yaw velocities during saccades (color code as in **D**). **(F)** Distribution of the time lapse between the velocity peak of head and body during the saccades.

**Figure 3:**
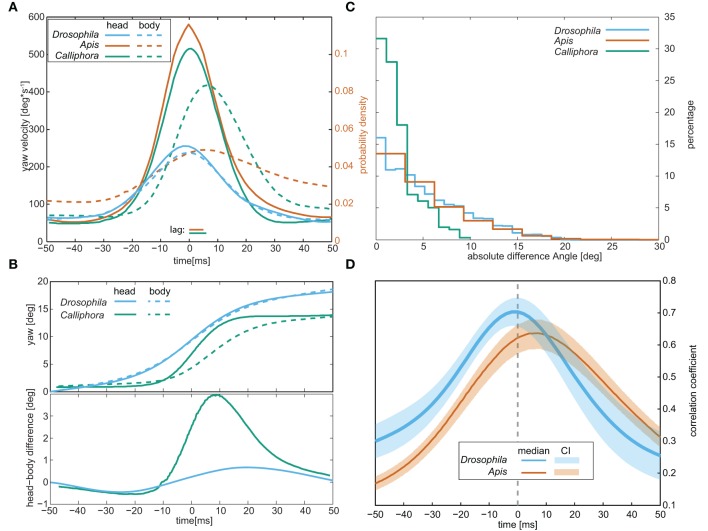
**Comparison of the head and body movements of walking *Drosophila*, walking blowflies (“*Calliphora”*), and flying honey bees (“*Apis”*)**. **(A)** Respective average yaw velocities during saccades. Note that for bees only the probability density is shown. **(B)** Average yaw angles (top) of the head and body and the difference between them (bottom) during saccadic body rotations. **(C)** Distributions of the angles between head and body (the ϕ-angles) observed during the entire trajectories, including saccades, and the trajectories between them. **(D)** Correlation coefficients between the yaw angles of the head and the body during the saccades. *Calliphora* is not included in this panel since no such data is available for this species. Data for *Apis* was taken from Boeddeker (2010) and data for Calliphora from Blaj and van Hateren (2004).

Insects cannot directly move their eyes; in order to change their gaze direction, they have to turn the head about the body or, alternatively, the body together with the head. Because of the small size of *Drosophila*, resolving the relative orientations of the head and the rest of the body requires imaging with a high spatial resolution, which is only possible within a confined space. We spatially confined the flies by letting them pass through an L-shaped labyrinth, which forced them to change their course by an angle of 90° (Figure 2B). Seven flies were filmed while each passed the labyrinth three times from either side. Respective head and thorax orientations were subsequently deduced by fitting the head and thorax with 3D templates (Figure 2A lower frame). Manual analysis of a total of 16,000 frames revealed that when turning in the labyrinth, the flies also performed saccades (Figures 2B–D). Three to six subsequent saccades were observed while the flies passed the 90° corner (Figure 2B). Body and head rotations performed in the labyrinth are similar to those seen in the circular arena (compare Figure 1D and Figure 2C). Moreover, averaged yaw velocities and yaw angles were virtually identical for thorax and head (Figures 2C), as were the respective distributions of the saccade amplitudes and angular velocities (Figures 2D,E). The temporal disparity between the peak velocities of head and body (Figure 2F) was about 2 ms for approximately 75% of the saccades. Hence, during the saccadic turns, the head does not move faster than the thorax, identifying the saccadic turns of walking *Drosophila* as pure body saccades in which the head moves together with the body. This saccadic behavior differs from the saccades of e.g., flying honeybees and walking blowflies, which rotate the head faster than the body (see Figure 3A), reducing the effective duration of the saccades (Blaj and van Hateren, 2004; Boeddeker, 2010): the different rotation speeds of head and body were seen when we extracted their respective yaw angles for blowflies from (Blaj and van Hateren, 2004), where the relative angles between head and body—the ϕ-angles—reach up to 4° (Figure 3B). In *Drosophila*, by contrast, the yaw-angles of the head superimposed with those of the body, yielding ϕ-angles of maximally 1° (Figure 3B).

Judging from the ϕ-angle distribution obtained for the whole labyrinth passage (Figure 3C), *Drosophila* is able to move its head about its body by up to 20°: the ϕ-angle distribution closely resembled those reported for flying honeybees (Boeddeker, 2010). According to Blaj (2004), the respective distribution for walking *Calliphora* is shifted toward smaller angles (maximum ϕ-angles around 10°), though recent studies (Kress and Egelhaaf, 2014a) suggest that larger ϕ-angles occur in this species as well. For *Drosophila*, we cross-correlated the yaw velocities of the head and body during the whole trajectory and found that it peaks at zero phase-lag, whereas the head leads the body by ca. 8 ms in flying honeybees (Boeddeker, 2010) (Figure 3D). Hence, unlike bees, walking *Drosophila* does not move its head relative to the body during saccadic body turns.

### Head movements could speed up saccades but would hardly change the retinal image

**Figure 4:**
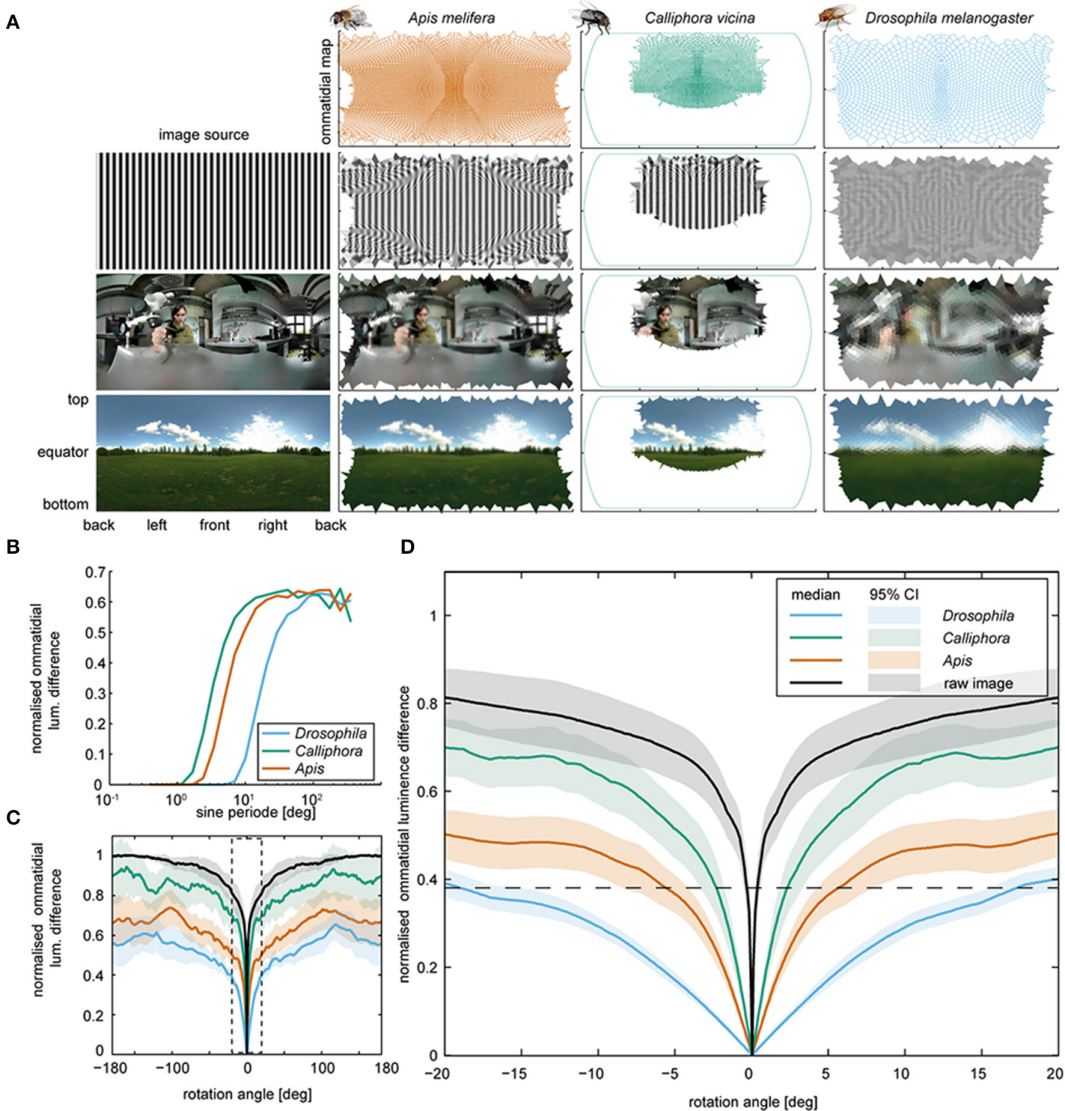
**Retinal inputs and image shifts. (A)** Voronoi cells computed for the eyes of *Drosophila, Apis*, and *Calliphora* (top row), and respective retinal images (subsequent rows) deduced for three different images (left). The image in the lower row was taken by Janne Voutilainen (for a complete list of the images used, see Supplemental Materials). Note that for *Calliphora*, only the central field of view is covered (for data sources, see Materials and Methods). **(B)** Mean ommatidial luminescence difference, caused by shifting sinusoidal stripe patterns with different sine periods by half a sine period. The differences were normalized to the maximal pixel wise difference of the original images and plotted against the sine period. **(C)** Mean ommatidial luminescence difference caused by shifting images depicting a naturalistic scenes in steps of 0.1° around the animal from −180 to +180° (for images, see Supplementary Figure 1). The differences were normalized to the corresponding pixel wise difference of the image and plotted against the rotation angles. Lines: means; transparent areas: 95% confidence intervals. A zoomed in version of the data in the dashed box is plotted in Figure 4D. For respective data obtained with artificial images with an 1/f spatial distribution, see Supplementary Figure 2). **(D)** Close-up from panel **C**. Note that *Drosophila* generates a smaller ommatidial luminescence difference by turning by more than 15° (as seen during the body saccades) than *Calliphora* generates by turning about 4° (as seen during a *Calliphora* head saccade).

Blowflies and honeybees surpass *Drosophila* in terms of visual acuity: the acceptance angle of *Drosophila* photoreceptors is about 10-fold larger (Laughlin and Horridge, 1971; Smakman et al., 1984; Gonzalez-Bellido et al., 2011), and the bee eye also comprises about 8 times more ommatidia than that of *Drosophila* (Buchner, 1971; Seidl, 1986; Dickson et al., 2008; Stürzl et al., 2010). Using available information about species-specific ommatidium numbers, positions, and orientations (Buchner, 1971; Seidl, 1986; Petrowitz et al., 2000; Dickson et al., 2008; Stürzl et al., 2010), we calculated how different panoramas are mapped onto the retinae of these insects. We modeled the field of view of each ommatidium by fitting Gaussians to published photoreceptor acceptance angles. As expected, Mercator plots of the predicted retinal inputs were more blurred for *Drosophila* than for blowflies and bees (Figure 4A). Using sinusoidal gratings with different angular frequencies as panoramas, we next determined how the input of each ommatidium changes when the panorama shifts vertically by half a wavelength (Figure 4B). This revealed that blowflies and bees should be able to optically resolve objects with a horizontal angular extension of 1° and *Drosophila* of 7°, consistent with experimental observations (Nordström and O'Carroll, 2006; Fox and Frye, 2014; Fox et al., 2014).

Using panoramic photographs of natural scenes (images courtesy of Janne Voutilainen and Aldo Hoeben) we determined the rotational image difference (Zeil, 2012) to assess how the contrast will change for each ommatidium when the head rotates (Figures 4C,D). For a head rotation of 4°, as observed in blowflies, we obtained relative contrast changes of the retinal image of ca. 48% for blowflies and 33.3% for honeybees. For *Drosophila*, we obtained a contrast change of only 14% for the same rotation (Figure 4D). To achieve a contrast change of 35%, *Drosophila* would have to turn its head by 15 instead of 4°. We also tested 10 panoramas with a 1/f^2^ frequency distribution (Field, 1987; van der Schaaf and van Hateren, 1996; Saremi and Sejnowski, 2013), which yielded quantitatively equivalent results (see Supplementary Figure 2). Hence, by turning its whole body, *Drosophila* generates the same contrast change that blowflies reach by solely rotating their head. In principle, active head rotations during saccadic body turns of *Drosophila* could reduce the rotation time, as is the case in blowfly and also honeybees (Blaj and van Hateren, 2004; Boeddeker, 2010). Due to the low spatial acuity of the *Drosophila* eye, however, such additional head rotations would result in minor contrast changes that the eye might not be able to detect.

### Absence of head movements associates with the absence of haltere oscillations

**Figure 5:**
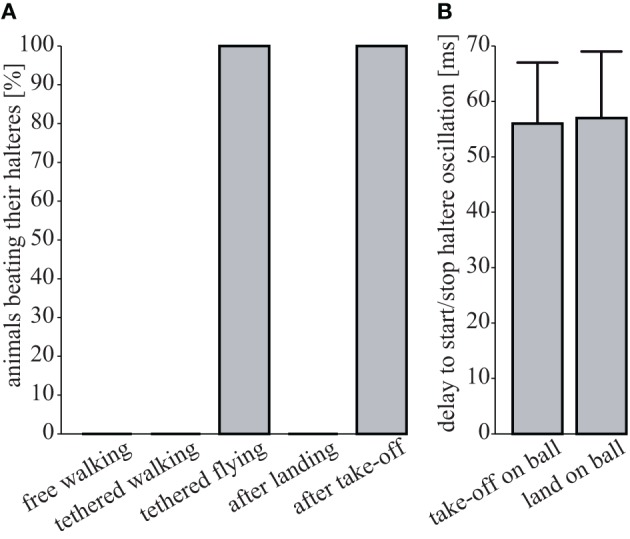
**Absence of haltere oscillations**. **(A)** Percentage of flies oscillating their halteres under different conditions (free walking, *N* = 9; tethered walking, *N* = 6; tethered flying, *N* = 6; after landing, *N* = 3; after take-off, *N* = 3). **(B)** Time delay between the last tarsus leaving the ball (“take off on ball”) or the first tarsus touching the ball (“landing on ball”) and the respective on- and offsets of haltere oscillations (*N* = 3 each, means ± 1 SD).

Head rotations in flying and walking blowflies are associated with active haltere vibrations (Nalbach and Hengstenberg, 1994; Haag et al., 2010), which continuously oscillate while the flies walk (Sandeman and Markl, 1980). We tested for such haltere oscillations in walking *Drosophila* and found that there are none. Haltere oscillations were observed only upon take-off and during flight, yet they immediately ceased upon landing (see Figure 5). Unlike walking blowflies, *Drosophila* neither performs head saccades nor does it oscillate its halteres while it walks.

## Discussion

The walking behavior of *Drosophila* imagines has been studied on various levels of complexity, ranging from molecular (Brierley et al., 2012; Baek et al., 2013; Bidaye et al., 2014; Desai et al., 2014) and neuronal mechanisms (Chiappe et al., 2010; Seelig et al., 2010; Ofstad et al., 2011; Ping et al., 2011) to leg movements (Strauß and Heisenberg, 1990; Kain et al., 2013; Wosnitza et al., 2013), walking trajectories (Mendes et al., 2013), and group behavior (Liu et al., 2011; Billeter et al., 2012; Hahn et al., 2013). Walking trajectories and movements have been studied in the context of object fixation, and walking was also shown to facilitate motion detection (Rosner et al., 2009; Chiappe et al., 2010). The need to detect motion is reportedly reflected by the locomotion behavior of flying *Drosophila* (Heisenberg and Wolf, 1979; Tammero and Dickinson, 2002; Bender and Dickinson, 2006; Censi et al., 2013; Muijres et al., 2014) and other dipteran species. For *Drosophila*, retinal object motion was also shown to serve as a cue for distance estimation (Schuster et al., 2002). Our analysis shows that walking *Drosophila* separate translational movements from rotational ones by performing saccadic turns. These turns are slower than those performed by flying *Drosophila*, with their duration resembling those reported for the saccadic rotations of walking blowflies and bees. We further show that additional, correlated head movements do not accompany the body saccades of walking *Drosophila*, documenting that, while walking, *Drosophila* lacks the head saccades that reduce the effective turning duration in blowflies and bees. This absence of head saccades seems to reflect optical constraints imposed by the eye, whose visual acuity is lower in *Drosophila* than in these other insect species. The absence of head saccades also associates with the absence of haltere oscillations. In blowflies, active haltere oscillations are implicated in the generation of head saccades, indicating that walking *Drosophila* lack both these head saccades and their haltere control.

In addition to facilitating distance estimation, the saccadic movement strategy used by *Drosophila* might reduce motion blur. Motion blur occurs when the speed by which the image changes exceeds the integration time of the photoreceptors and reduces the perceived image contrast. In insects, motion blur sets in when the stimulus travels faster than one ommatidial acceptance angle (Δρ in deg.) during the integration time of the photoreceptors (Δt in s) (Land, 2003). Photoreceptor integration times Δt are about 20 ms for *Drosophila* (Juusola, 2000; Hardie, 2001; Niven et al., 2004) and 5–10 ms for *Calliphora* (Tatler et al., 2000). The respective ommatidial acceptance angles Δρ for the two species are 10 (*Drosophila*: Gonzalez-Bellido et al., 2011) and 1° (*Calliphora*: Smakman et al., 1984). These figures translate into motion blur thresholds of 500 deg^*^s^−1^ for *Drosophila* and 100–200 deg^*^s^−1^ for *Calliphora*. To avoid motion blur, head stabilization thus seems less critical for *Drosophila* than for *Calliphora*.

According to our analysis, walking *Drosophila*, like flying ones, separate rotations from translations to facilitate motion vision. During walking, however, this separation seems incomplete. During translations, substantial rotations persist, including head rotations with angles of up to 20° relative to the body. Head rotations during translations have also been observed in flying bees (Boeddeker, 2010) and walking blowflies (Blaj and van Hateren, 2004; Kress and Egelhaaf, 2014a), and *Drosophila* during flight (Fox and Frye, 2014). In walking blowflies, these head rotations reportedly arise from the walking apparatus and depend on the substrate the animal is walking on (Kress and Egelhaaf, 2012, 2014a), and in tethered *Drosophila* they were implicated in wide-field image fixation (Fox and Frye, 2014). Judging from our data, about half of the head turning angles of walking *Drosophila* lie below 5°, causing retinal image shifts of less than fifteen percent. Given these rather marginal image shifts, one might speculate that flying *Drosophila* also do not perform head-saccades, although their halteres oscillate.

## Author contributions

Bart Geurten and Martin Göpfert designed the study. Kristina Corthals and Bart Geurten conducted the experiments. Philipp Jähde constructed the 3D templates used in manual tracing. Philipp Jähde, Kristina Corthals and Bart Geurten analyzed the trajectories. Bart Geurten analyzed, modeled and plotted the data. Martin Göpfert and Bart Geurten wrote the manuscript with intellectual contributions from all other authors.

## Funding statements

This work was supported by the Collaborative Research Center SFB 889 of the German Science Foundation (project C2, to Martin C. Göpfert).

### Conflict of interest statement

The authors declare that the research was conducted in the absence of any commercial or financial relationships that could be construed as a potential conflict of interest.

## References

[B1] Baek M. Enriquez J. Mann R. S. (2013). Dual role for Hox genes and Hox co-factors in conferring leg motoneuron survival and identity in *Drosophila*. Development 140, 2027–2038. 10.1242/dev.090902 23536569 PMC3631975

[B2] Bender J. A. Dickinson M. H. (2006). A comparison of visual and haltere-mediated feedback in the control of body saccades in *Drosophila melanogaster*. J. Exp. Biol. 209, 4597–4606. 10.1242/jeb.02583 17114395

[B3] Bidaye S. S. Machacek C. Wu Y. Dickson B. J. (2014). Neuronal control of *Drosophila* walking direction. Science 344, 97–101. 10.1126/science.1249964 24700860

[B4] Billeter J.-C. Jagadeesh S. Stepek N. Azanchi R. Levine J. D. (2012). *Drosophila melanogaster* females change mating behaviour and offspring production based on social context. Proc. Biol. Sci. 279, 2417–2425. 10.1098/rspb.2011.2676 22298851 PMC3350680

[B81] Blaj G. (2004). Walking and Vision in Blowflies. Ph.D. dissertation, University of Groningen.

[B5] Blaj G. van Hateren J. H. (2004). Saccadic head and thorax movements in freely walking blowflies. J. Comp. Physiol. A Neuroethol. Sens. Neural Behav. Physiol. 190, 861–868. 10.1007/s00359-004-0541-4 15278400

[B6] Boeddeker N. (2010). The fine structure of honeybee head and body yaw movements in a homing task. Proc. R. Soc. B Biol. Sci. 277, 1899–1906. 10.1098/rspb.2009.2326 20147329 PMC2871881

[B7] Braun E. Geurten B. Egelhaaf M. (2010). Identifying prototypical components in behaviour using clustering algorithms. PLoS ONE 5:e9361. 10.1371/journal.pone.0009361 20179763 PMC2825265

[B8] Brierley D. J. Rathore K. VijayRaghavan K. Williams D. W. (2012). Developmental origins and architecture of *Drosophila* leg motoneurons. J. Comp. Neurol. 520, 1629–1649. 10.1002/cne.23003 22120935

[B9] Buchner E. (1971). Dunkelanregung des Stationären Flugs der Fruchtfliege Drosophila. Ph.d. disscustion, University of Tübingen.

[B11] Buelthoff H. Poggio T. Wehrhahn C. (1980). 3-D analysis of flight trajectories of flies (*Drosophila melanogaster*). Z. Naturforsch. 35c, 811–845.

[B12] Censi A. Straw A. D. Sayaman R. W. Murray R. M. Dickinson M. H. (2013). Discriminating external and internal causes for heading changes in freely flying *Drosophila*. PLoS Comput. Biol. 9:e1002891. 10.1371/journal.pcbi.1002891 23468601 PMC3585425

[B13] Chiappe M. E. Seelig J. D. Reiser M. B. Jayaraman V. (2010). Walking modulates speed sensitivity in *Drosophila* motion vision. Curr. Biol. 20, 1470–1475. 10.1016/j.cub.2010.06.072 20655222 PMC4435946

[B14] Collett T. S. Land M. F. (1975). Visual spatial memory in a hoverfly. J. Comp. Physiol. 100, 59–84 10.1007/BF00623930

[B15] Desai B. S. Chadha A. Cook B. (2014). The stum gene is essential for mechanical sensing in proprioceptive neurons. Science 343, 1256–1259. 10.1126/science.1247761 24626929

[B16] Dickson W. B. Straw A. D. Dickinson M. H. (2008). Integrative model of *Drosophila* flight. AIAA J. 46, 2150–2164.

[B17] Dickson W. B. Straw A. D. Poelma C. Dickinson M. H. (2006). An integrative model of insect flight control, in 44th AIAA Aerospace Sciences Meeting and Exhibit (Reno, NV: American Institute of Aeronautics and Astronautics).

[B19] Field D. J. (1987). Relations between the statistics of natural images and the response properties of cortical cells. J. Opt. Soc. Am. A 4:2379. 10.1364/JOSAA.4.002379 3430225

[B20] Fox J. L. Aptekar J. W. Zolotova N. M. Shoemaker P. A. Frye M. A. (2014). Figure-ground discrimination behavior in *Drosophila*. I. Spatial organization of wing-steering responses. J. Exp. Biol. 217, 558–569. 10.1242/jeb.097220 24198267 PMC3922833

[B21] Fox J. L. Frye M. A. (2014). Figure-ground discrimination behavior in *Drosophila*. II. Visual influences on head movement. J. Exp. Biol. 217, 570–579. 10.1242/jeb.080192 24198264 PMC3922834

[B22] Fry S. N. Sayaman R. Dickinson M. H. (2003). The aerodynamics of free-flight maneuvers in *Drosophila*. Science 300, 495–498. 10.1126/science.1081944 12702878

[B24] Geiger G. Poggio T. (1977). On head and body movements of flying flies. Biol. Cybern. 25, 177–180 10.1007/BF00365214

[B25] Geurten B. R. H. Kern R. Braun E. Egelhaaf M. (2010). A syntax of hoverfly flight prototypes. J. Exp. Biol. 213, 2461–2475. 10.1242/jeb.036079 20581276

[B26] Gibson J. Olum P. Rosenblatt F. (1955). Parallax and perspective during aircraft landings. Am. J. Psychol. 68, 372–385. 10.2307/1418521 13248971

[B28] Gonzalez-Bellido P. T. Wardill T. J. Juusola M. (2011). Compound eyes and retinal information processing in miniature dipteran species match their specific ecological demands. Proc. Natl. Acad. Sci. U.S.A. 108, 4224–4229. 10.1073/pnas.1014438108 21368135 PMC3054003

[B29] Haag J. Wertz A. Borst A. (2010). Central gating of fly optomotor response. Proc. Natl. Acad. Sci. U.S.A. 107, 20104–20109. 10.1073/pnas.1009381107 21045125 PMC2993391

[B30] Hahn N. Geurten B. Gurvich A. Piepenbrock D. Kästner A. Zanini D.. (2013). Monogenic heritable autism gene neuroligin impacts *Drosophila* social behaviour. Behav. Brain Res. 252, 450–457. 10.1016/j.bbr.2013.06.020 23792025

[B31] Hardie R. C. (2001). Phototransduction in *Drosophila melanogaster*. J. Exp. Biol. 204, 3403–3409. 11707492 10.1242/jeb.204.20.3403

[B32] Heisenberg M. Wolf R. (1979). On the Fine Structure of Yaw Torque in Visual Flight Orientation of *Drosophila melanogaster* M. J. Comp. Physiol. A 130, 113–130 10.1007/BF00611046

[B33] Hofmann V. Geurten B. R. H. Sanguinetti-Scheck J. I. Gómez-Sena L. Engelmann J. (2014). Motor patterns during active electrosensory acquisition. Front. Behav. Neurosci. 8:186. 10.3389/fnbeh.2014.00186 24904337 PMC4036139

[B34] Juusola M. (2000). Light adaptation in *Drosophila* photoreceptors: I. response dynamics and signaling efficiency at 25degrees C. J. Gen. Physiol. 117, 3–25. 10.1085/jgp.117.1.3 11134228 PMC2232468

[B35] Kain J. Stokes C. Gaudry Q. Song X. Foley J. Wilson R.. (2013). Leg-tracking and automated behavioural classification in *Drosophila*. Nat. Commun. 4, 1910. 10.1038/ncomms2908 23715269 PMC3674277

[B37] Koenderink J. J. Doorn A. J. (1987). Facts on optic flow. Biol. Cybern. 56, 247–254. 10.1007/BF00365219 3607100

[B38] Kral K. Poteser M. (1997). Motion parallax as a source of distance information in locusts and mantids. J. Ins. Behav. 1, 145–163 10.1007/BF02765480

[B39] Kress D. Egelhaaf M. (2012). Head and body stabilization in blowflies walking on differently structured substrates. J. Exp. Biol. 215, 1523–1532. 10.1242/jeb.066910 22496289

[B40] Kress D. Egelhaaf M. (2014a). Gaze characteristics of freely walking blowflies in a goal-directed task. J. Exp. Biol. 217, 3209–3220. 10.1242/jeb.097436 25013104

[B41] Kress D. Egelhaaf M. (2014b). Impact of stride-coupled gaze shifts of walking blowflies on the neuronal representation of visual targets. Front. Behav. Neurosci. 8:00307. 10.3389/fnbeh.2014.00307 25309362 PMC4164030

[B43] Land M. F. (1973). Head movement of flies during visually guided flight. Nature 243, 299–300. 10.1038/243299a021543620

[B42] Land M. F. (1992). Visual tracking and pursuit: humans and arthropods compared. J. Insect Physiol. 38, 939–951 10.1016/0022-1910(92)90002-U

[B44] Land M. F. (1999). Motion and vision: why animals move their eyes. J. Comp. Physiol. A Neuroethol. Sens. Neural Behav. Physiol. 185, 341–352. 10.1007/s003590050393 10555268

[B45] Land M. F. (2003). Visual Acuity in Insects. Annu. Rev. Entomol. 42, 147–177. 10.1146/annurev.ento.42.1.147 15012311

[B46] Laughlin S. B. Horridge G. A. (1971). Angular sensitivity of the retinula cells of dark-adapted worker bee. Zeitschrift für Vergleichende Physiol. 74, 329–335 10.1007/BF00297733

[B27] Lejeune Dirichlet G. (1850). Über die Reduktion der positiven quadratischen Formen mit drei unbestimmten ganzen Zahlen. J. für die Reine und Angew. Math. 40, 209–227 10.1515/crll.1850.40.209

[B47] Liu W. Liang X. Gong J. Yang Z. Zhang Y.-H. Zhang J.-X.. (2011). Social regulation of aggression by pheromonal activation of Or65a olfactory neurons in *Drosophila*. Nat. Neurosci. 14, 896–902. 10.1038/nn.2836 21685916

[B48] MacQueen J. B. (1967). Some methods for classification and analysis of multivariate observations. Proc. 5th Berkeley Symp. Math. Stat. Probab. 1, 281–297.

[B49] Mendes C. S. Bartos I. Akay T. Márka S. Mann R. S. (2013). Quantification of gait parameters in freely walking wild type and sensory deprived *Drosophila melanogaster*. Elife 2:e00231. 10.7554/eLife.00231 23326642 PMC3545443

[B50] Milligan G. W. Cooper M. C. (1987). Methodology review: clustering methods. Appl. Psychol. Meas. 11, 329–354 10.1177/014662168701100401

[B51] Muijres F. T. Elzinga M. J. Melis J. M. Dickinson M. H. (2014). Flies evade looming targets by executing rapid visually directed banked turns. Science 344, 172–177. 10.1126/science.1248955 24723606

[B52] Murtagh F. Contreras P. (2012). Algorithms for hierarchical clustering: an overview. Wiley Interdiscip. Rev. Data Min. Knowl. Discov. 2, 86–97 10.1002/widm.53

[B53] Nalbach G. Hengstenberg R. (1994). The halteres of the blowfly *Calliphora*. J. Comp. Physiol. A 175, 695–708 10.1007/BF00191842

[B54] Niven J. E. Vähäsöyrinki M. Juusola M. French A. S. (2004). Interactions between light-induced currents, voltage-gated currents, and input signal properties in *Drosophila* photoreceptors. J. Neurophysiol. 91, 2696–2706. 10.1152/jn.01163.2003 14749305

[B55] Nordström K. O'Carroll D. C. (2006). Small object detection neurons in female hoverflies. Proc. Biol. Sci. 273, 1211–1216. 10.1098/rspb.2005.3424 16720393 PMC1560283

[B56] Ofstad T. A. Zuker C. S. Reiser M. B. (2011). Visual place learning in *Drosophila melanogaster*. Nature 474, 204–207. 10.1038/nature10131 21654803 PMC3169673

[B57] Petrowitz R. Dahmen H. Egelhaaf M. Krapp H. G. (2000). Arrangement of optical axes and spatial resolution in the compound eye of the female blowfly *Calliphora*. J. Comp. Physiol. A 186, 737–746. 10.1007/s003590000127 11016789

[B58] Ping Y. Waro G. Licursi A. Smith S. Vo-Ba D.-A. Tsunoda S. (2011). Shal/K(v)4 channels are required for maintaining excitability during repetitive firing and normal locomotion in *Drosophila*. PLoS ONE 6:e16043. 10.1371/journal.pone.0016043 21264215 PMC3022017

[B59] Ribak G. Egge A. R. Swallow J. G. (2009). Saccadic head rotations during walking in the stalk-eyed fly (*Cyrtodiopsis dalmanni*). Proc. Biol. Sci. 276, 1643–1649. 10.1098/rspb.2008.1721 19203925 PMC2660984

[B60] Rosner R. Egelhaaf M. Grewe J. Warzecha A. K. (2009). Variability of blowfly head optomotor responses. J. Exp. Biol. 212, 1170–1184. 10.1242/jeb.027060 19329750

[B61] Sandeman D. C. Markl H. (1980). Head movements in flies (*Calliphora*) produced by deflexion of the halteres. J. Exp. Biol. 85, 43–60.

[B62] Saremi S. Sejnowski T. J. (2013). Hierarchical model of natural images and the origin of scale invariance. Proc. Natl. Acad. Sci. U.S.A. 110, 3071–3076. 10.1073/pnas.1222618110 23382241 PMC3581899

[B65] Schilstra C. van Hateren J. H. (1998). Stabilizing gaze in flying blowflies. Nature 395, 654. 10.1038/27114 9790186

[B66] Schuster S. Strauss R. Götz K. (2002). Virtual-reality techniques resolve the visual cues used by fruit flies to evaluate object distances. Curr. Biol. 18, 1591–1594. 10.1016/S0960-9822(02)01141-7 12372251

[B67] Seelig J. D. Chiappe M. E. Lott G. K. Dutta A. Osborne J. E. Reiser M. B.. (2010). Two-photon calcium imaging from head-fixed *Drosophila* during optomotor walking behavior. Nat. Methods 7, 535–540. 10.1038/nmeth.1468 20526346 PMC2945246

[B68] Seidl R. (1986). Die Sehfelder und Ommatidien-Divergenzwinkel von Arbeiterin, Königin und Drohn der Honigbiene (Apis mellifica). Darmstadt: Technische Hochschule.

[B69] Smakman J. G. J. Hateren J. H. Stavenga D. G. (1984). Angular sensitivity of blowfly photoreceptors: intracellular measurements and wave-optical predictions. J. Comp. Physiol. A 155, 239–247 10.1007/BF00612641

[B70] Stanford T. R. Shankar S. Massoglia D. P. Costello M. G. Salinas E. (2010). Perceptual decision making in less than 30 milliseconds. Nat. Neurosci. 13, 379–385. 10.1038/nn.2485 20098418 PMC2834559

[B71] Strauß R. Heisenberg M. (1990). Coordination of legs during straight walking and turning in *Drosophila melanogaster*. J. Comp. Physiol. A 167, 403–412. 2121965 10.1007/BF00192575

[B72] Stürzl W. Boeddeker N. Dittmar L. Egelhaaf M. (2010). Mimicking honeybee eyes with a 280 degrees field of view catadioptric imaging system. Bioinspir. Biomim. 5:036002. 10.1088/1748-3182/5/3/036002 20689158

[B73] Tammero L. F. Dickinson M. H. (2002). The influence of visual landscape on the free flight behavior of the fruit fly *Drosophila melanogaster*. J. Exp. Biol. 205, 327–343. 11854370 10.1242/jeb.205.3.327

[B74] Tatler B. O'Carroll D. C. Laughlin S. B. (2000). Temperature and the temporal resolving power of fly photoreceptors. J. Comp. Physiol. A Neuroethol. Sens. Neural Behav. Physiol. 186, 399–407. 10.1007/s003590050439 10798727

[B63] van der Schaaf A. van Hateren J. H. (1996). Modelling the power spectra of natural images: statistics and information. Vision Res. 36, 2759–2770. 10.1016/0042-6989(96)00002-8 8917763

[B75] Voronoi G. (1908). Nouvelles applications des paramètres continus à la théorie des formes quadratiques. Premier mémoire. Sur quelques propriétés des formes quadratiques positives parfaites. J. für die reine und Angew. Math. (Crelle's J.) 1908, 97–102.

[B76] Wosnitza A. Bockemühl T. Dübbert M. Scholz H. Büschges A. (2013). Inter-leg coordination in the control of walking speed in *Drosophila*. J. Exp. Biol. 216, 480–491. 10.1242/jeb.078139 23038731

[B77] Zeil J. (1986). The territorial flight of male houseflies (*Fannia canicularis* L.). Behav. Ecol. Sociobiol. 19, 213–219 10.1007/BF00300862

[B78] Zeil J. (1993). Orientation flights of solitary wasps (*Cerceris; Sphecidae; Hymenoptera*). J. Comp. Physiol. A. 172, 207–222 10.1007/BF00189397

[B79] Zeil J. (1996). The control of optic flow during learning flights. J. Comp. Physiol. A. 180, 25–37 10.1007/s003590050024

[B80] Zeil J. (2012). Visual homing: an insect perspective. Curr. Opin. Neurobiol. 22, 285–293. 10.1016/j.conb.2011.12.008 22221863

