# Supplementary material for: Saccadic body turns in walking Drosophila
Source: Front Behav Neurosci. 2014 Oct 22;8:365. doi: 10.3389/fnbeh.2014.00365 (PMC4205811; doi:10.3389/fnbeh.2014.00365)
Supplement: Supplementary file 1 [file Image1.PDF]

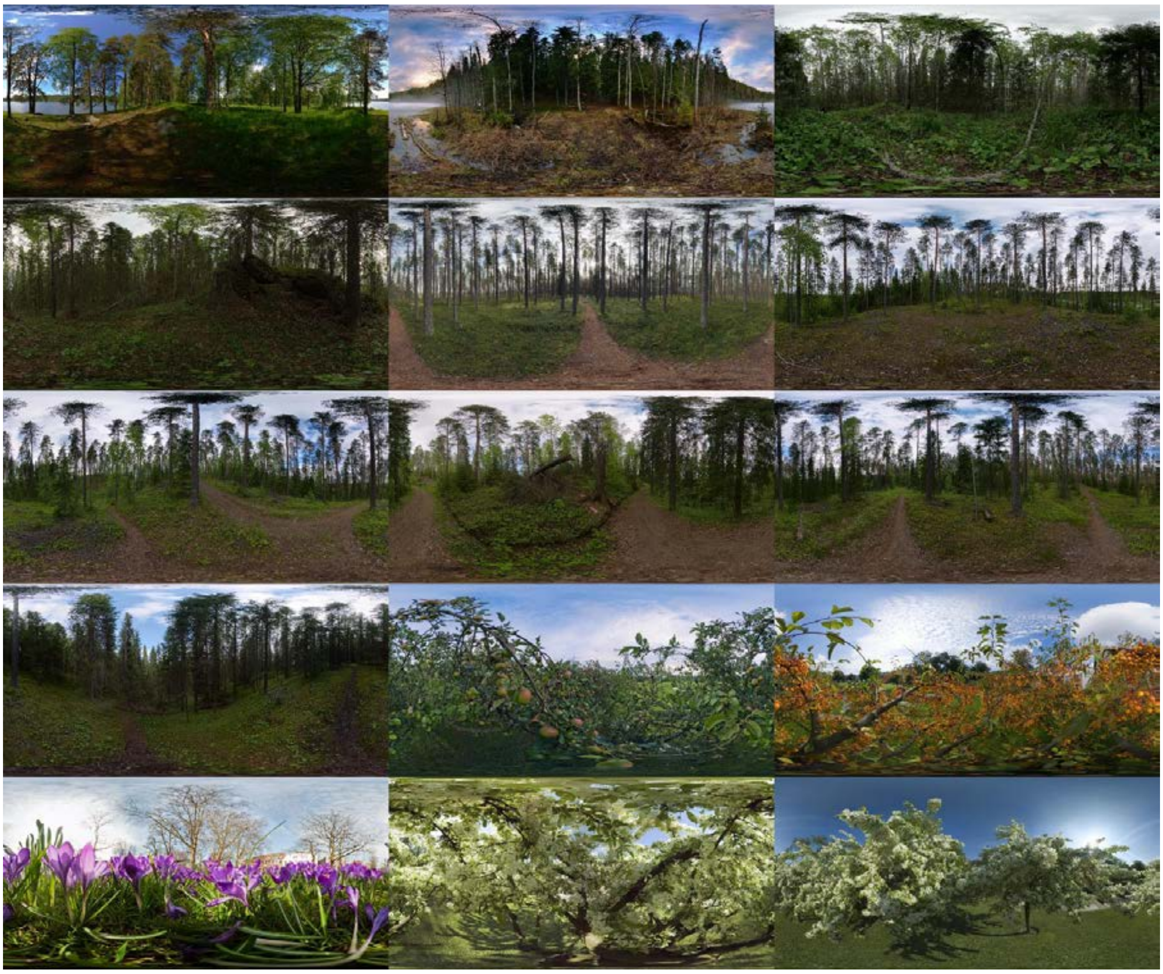

Supplementary Figure 1. Natural scenes used for generating the ommatidial luminescence differences in Fig. 4 C, D. Each image was tested at a resolution of 3600x 1800 pixel. The 10 forest scenes were photographed by Janne Voutilainen, and the 5 close-ups of different plants by Aldo Hoeben. All pictures are licensed under creative commons and the respective artists were asked for permission. For more artwork, see Janne Voutilainen's homepage at https://www.flickr.com/photos/jannefoo and Aldo Hoeben's homepage at https://www.flickr.com/photos/82678249@N00/.
