# Supplementary material for: Saccadic body turns in walking Drosophila
Source: Front Behav Neurosci. 2014 Oct 22;8:365. doi: 10.3389/fnbeh.2014.00365 (PMC4205811; doi:10.3389/fnbeh.2014.00365)
Supplement: Supplementary file 2 [file Image2.PDF]

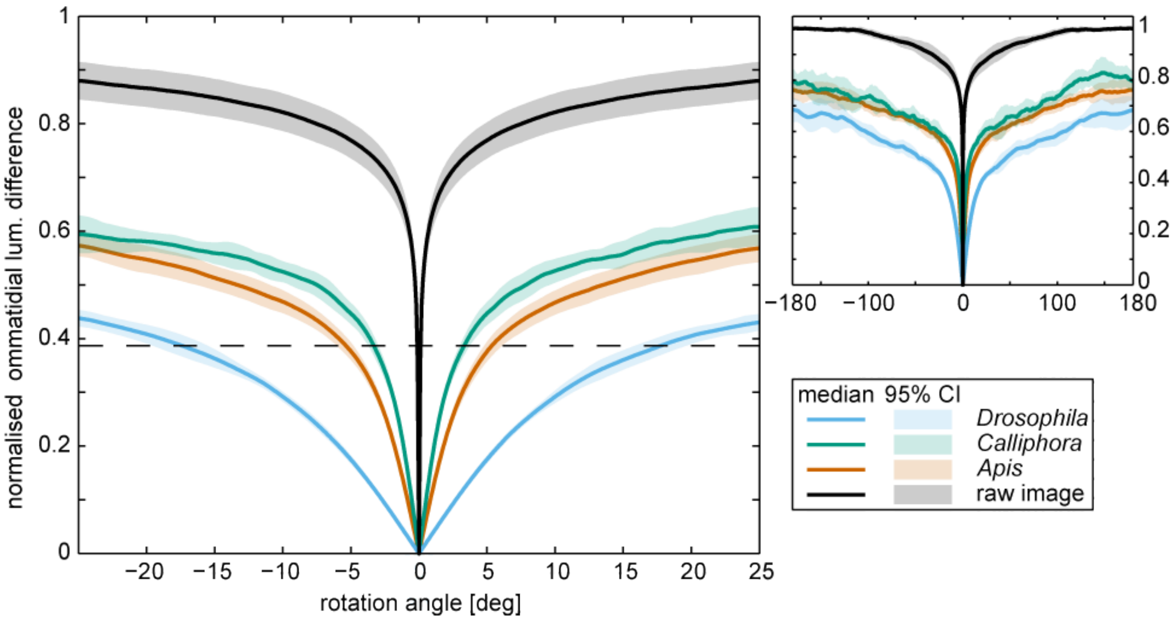

Supplementary Figure 2. Ommatidial luminescence differences as depicted in Fig. 4C, D obtained for images with a 1/f spatial frequency distribution. Note the close correspondence with Fig. 4C,D.
